# MAP4K4 promotes pancreatic tumorigenesis via phosphorylation and activation of mixed lineage kinase 3

**DOI:** 10.1038/s41388-021-02007-w

**Published:** 2021-09-13

**Authors:** Sunil Kumar Singh, Sandeep Kumar, Navin Viswakarma, Daniel R. Principe, Subhasis Das, Gautam Sondarva, Rakesh Sathish Nair, Piush Srivastava, Subhash C. Sinha, Paul J. Grippo, Gregory R. J. Thatcher, Basabi Rana, Ajay Rana

**Affiliations:** 1 Department of Surgery, Division of Surgical Oncology, the University of Illinois at Chicago, Chicago, IL 60612 USA; 2 Weill Cornell Medicine, New York, NY 10021 USA; 3 Department of Medicine, the University of Illinois at Chicago, Chicago, IL 60612 USA; 4 Department of Pharmacology and Toxicology, University of Arizona, Tucson, AZ 85721 USA; 5 University of Illinois Hospital & Health Sciences System Cancer Center, the University of Illinois at Chicago, Chicago, IL 60612 USA; 6 Jesse Brown VA Medical Center, Chicago, IL 60612 USA

**Keywords:** Pancreatic cancer, Stress signalling

## Abstract

MAP4K4 is a Ste20 member and reported to play important roles in various pathologies, including in cancer. However, the mechanism by which MAP4K4 promotes pancreatic cancer is not fully understood. It is suggested that MAP4K4 might function as a cancer promoter via specific downstream target(s) in an organ-specific manner. Here we identified MLK3 as a direct downstream target of MAP4K4. The MAP4K4 and MLK3 associates with each other, and MAP4K4 phosphorylates MLK3 on Thr738 and increases MLK3 kinase activity and downstream signaling. The phosphorylation of MLK3 by MAP4K4 promotes pancreatic cancer cell proliferation, migration, and colony formation. Moreover, MAP4K4 is overexpressed in human pancreatic tumors and directly correlates with the disease progression. The MAP4K4-specific pharmacological inhibitor, GNE-495, impedes pancreatic cancer cell growth, migration, induces cell death, and arrests cell cycle progression. Additionally, the GNE-495 reduced the tumor burden and extended survival of the KPC mice with pancreatic cancer. The MAP4K4 inhibitor also reduced MAP4K4 protein expression, tumor stroma, and induced cell death in murine pancreatic tumors. These findings collectively suggest that MLK3 phosphorylation by MAP4K4 promotes pancreatic cancer, and therefore therapies targeting MAP4K4 might alleviate the pancreatic cancer tumor burden in patients.

## Introduction

Mitogen-Activated Protein Kinase Kinase Kinase Kinase 4 (MAP4K4), also known as HGK (hematopoietic progenitor kinase/germinal center kinase-like kinase), is a serine/threonine kinase and belongs to the Ste20 family of kinases [1, 2]. The functional significance of MAP4K4 in normal embryonic development is evident because the whole body [3] or endothelial-specific knockout animals do not survive [4]. Besides its indispensable function in embryonic development, MAP4K4 plays a central role in systemic inflammation [5], lung inflammation [6], focal adhesion dynamic [7], insulin sensitivity [8], atherosclerosis [4], immunity [9], and cancer [10–13]. It has been suggested that MAP4K4 could contribute to oncogenic transformation through regulating downstream targets [10]. MAP4K4 phosphorylates TRAF2 in T cells, promoting its degradation [14]; however, this has not been implicated in cancer. MAP4K4 also directly phosphorylates a pleckstrin domain-containing protein FARP1 [15]; however, the implication of this phosphorylation in cancer is unknown. The information about the regulation of MAP4K4 by natural stimuli is similarly limited, and TNFα is reported as a bonafide agonist of MAP4K4 [16, 17]. The TNFα is known to induce inflammation and cancer; however, whether TNFα-induced MAP4K4 promotes cancer directly or indirectly is unclear [10]. We sought to determine the MAP4K4-induced downstream signaling that can potentially promote oncogenic transformation.

Earlier we reported that a MAP3K member, MLK3, is activated by TNFα [18]. Considering that MAP4K4 is a Ste20 member and MLK3 is a MAP3K member and both are activated by a common agonist, TNFα; we planned to determine any possible functional interaction between these two kinases and define the downstream signaling. MLK3 is a member of a MAP3K family called MLKs, which are unique because they contain signature sequences of both Ser/Thr and Tyr kinases in their catalytic domain [19, 20]. Out of all the MLK members, MLK3 has widely been explored [19–22], and we reported earlier that it plays a role in breast cancer [21]. We also reported that MLK3 kinase activity and function are regulated by estrogen in ER^ +^ breast cancer [21] and through HER2 amplification in HER2^+^ breast cancer [23]. Since we observed that MLK3 and MAP4K4 are overexpressed in pancreatic cell lines and tumors, and TNFα (a common agonist) can promote pancreatic cancer [24, 25], we planned to elucidate the functions of the MAP4K4-MLK3 axis in pancreatic cancer models.

Here we demonstrate that endogenous MAP4K4 and MLK3 proteins interact with each other, and MAP4K4 is an upstream regulator of MLK3 that modulates MLK3 kinase activity and function via direct phosphorylation of a specific Threonine (Thr) residue (i.e., T738). The MLK3 phosphorylation by MAP4K4 consequently promotes pancreatic cancer cell proliferation, colony formation, and cell migration. Interestingly, the MAP4K4 expression positively correlates with the pancreatic cancer tumor grades. We used MAP4K4-specific pharmacological inhibitor GNE-495 [26] that induced cell death and blocked cell cycle progression and migration of pancreatic cancer cells. Moreover, the knockdown of MAP4K4 in PDAC cells significantly induced cell death and blocked cell proliferation, similar to GNE-495. The GNE-495 significantly extended the survival and reduced pancreatic cancer tumor burden in the KPC mouse model of pancreatic ductal adenocarcinoma (PDAC). The cancerous lesions and ductal cell proliferation in mouse pancreatic tissues were significantly reduced, and there was substantial cell death in the neoplastic pancreatic ductal cells by GNE-495. Our data suggest that MLK3 is a direct downstream substrate of MAP4K4 that supports pancreatic cancer development via phosphorylating MLK3.

## Results

### MAP4K4 associates with and regulates MLK3 in the TNFα-induced JNK activation pathway

**Fig. 1:**
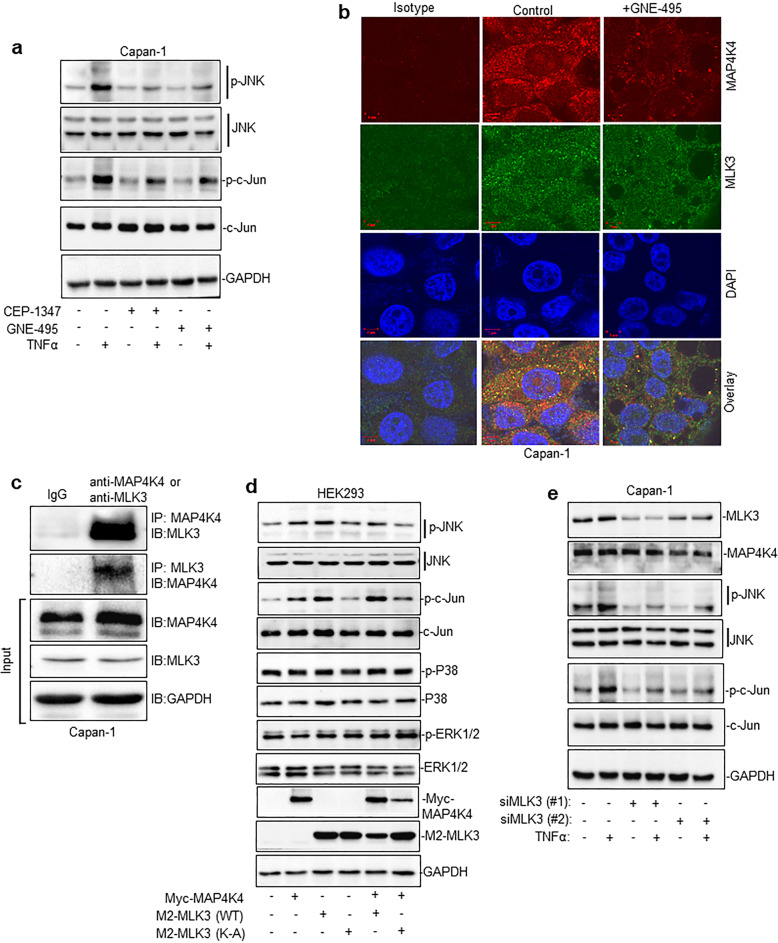
MAP4K4 associates with MLK3 and regulates downstream signaling in PDAC. **a** Capan-1 cells were pretreated either with MAP4K4 inhibitor, GNE-495 (2 µM) or MLKs inhibitor, CEP-1347 (500 nM) for 24 h. in presence or absence of TNFα (5 ng for 15 min), and cell extracts were blotted with: anti- p-JNK, JNK, p-cJun, cJun, and GAPDH antibodies. **b** Confocal microscopy images of endogenous MAP4K4 and MLK3 proteins co-localization in presence and absence of GNE-495, in Capan-1 cells, Scale bar, 5 µm. **c** Endogenous MAP4K4 or MLK3 from Capan-1 cells were immunoprecipitated and blotted either with anti-MLK3 or anti-MAP4K4 antibodies to determine their interaction. **d** HEK-293 cells were transfected with the indicated expression vectors of Myc-tagged MAP4K4 and Flag-tagged MLK3 (WT) or kinase inactive, MLK3 (K-A), and cell extracts were blotted with indicated antibodies. **e** MLK3 was knockdown using two specific siRNAs and treated with TNFα (5 ng for 15 min), the cell extracts were blotted with indicated antibodies. The western blot and microscopy analyses presented; *n* = 3.

The pro-inflammatory cytokine, TNFα, is reported to activate MAP4K4 [16, 17] as well as MLK3 [18], and activation of both the kinases reportedly activate JNK activity [16, 18, 19]. To explore the potential functional relation between MAP4K4 and MLK3, we used MAP4K4-specific inhibitor, GNE-495 [26], and MLK3 inhibitor, CEP-1347 [27], and determined their effects on TNFα-induced activation of JNK and its downstream target, c-JUN in PDAC cell lines. The TNFα was able to activate JNK and c-Jun as determined by their phosphorylation (Fig. 1a and Supplementary Fig. 1a), whereas both GNE-495 and CEP-1347 attenuated the TNFα-induced activation of JNK and c-JUN in Capan-1 (Fig. 1a) and PANC-1 (Supplementary Fig. 1a) cell lines. The selection of PDAC cell lines was based on the relative expressions of both the MAP4K4 and MLK3 proteins (Supplementary Fig. 1b). MAP4K4 was highly expressed in Capan-1 cells where MLK3 was also present in substantial amounts (Supplementary Fig. 1b) and therefore, we determined the co-localization of these two proteins in Capan-1 cell line. There was a clear indication of co-localization of MAP4K4 and MLK3 in the cytoplasm of Capan-1 cells (Fig. 1b). Interestingly, MAP4K4 inhibitor, GNE-495 reduced the expression of both the MAP4K4 and MLK3 proteins and similarly the co-localization of both the proteins in the cytoplasm was also reduced (Fig. 1b). Next, we examined endogenous interaction between MAP4K4 and MLK3 proteins by co-immunoprecipitation. The immunoprecipitation of either MAP4K4 or MLK3, showed that these two proteins do interact with each other (Fig. 1c and Supplementary Fig.1c). Next, we determined whether MAP4K4 or MLK3 is upstream kinase, and which of the downstream MAPK pathways are cooperatively regulated by MAP4K4 and MLK3. The human embryonic kidney, HEK-293 cells were transfected with either MAP4K4 along with MLK3 -WT or -MLK3 (K-A) [kinase inactive]. The cell extracts were blotted with phospho-antibodies against JNK, c-Jun, p38, and ERK1/2. These extracts were also blotted with their corresponding antibodies that recognize their total expressions. Interestingly, the kinase-dead, MLK3 (K-A) significantly blocked MAP4K4-induced JNK activation, which was also evident by the reduced p-c-Jun signals (Fig. 1d). However, the activations of neither p38 nor ERK1/2 MAPKs were affected by MAP4K4 or MLK3, either alone or together. To further confirm that MLK3 is the downstream target of MAP4K4, we knocked down MLK3 in Capan-1 cells by using MLK3-specific siRNAs and treated either with vehicle or the common agonist of MLK3 and MAP4K4, TNFα. The p-JNK and p-c-Jun signals were downregulated in cells where MLK3 was knocked down (Fig. 1e). Taken together, our results demonstrate that the MAP4K4 and MLK3 do interact, and MAP4K4 acts as an upstream regulator of MLK3-JNK axis.

### MAP4K4 phosphorylates MLK3 to increase its kinase activity and downstream signaling

**Fig. 2:**
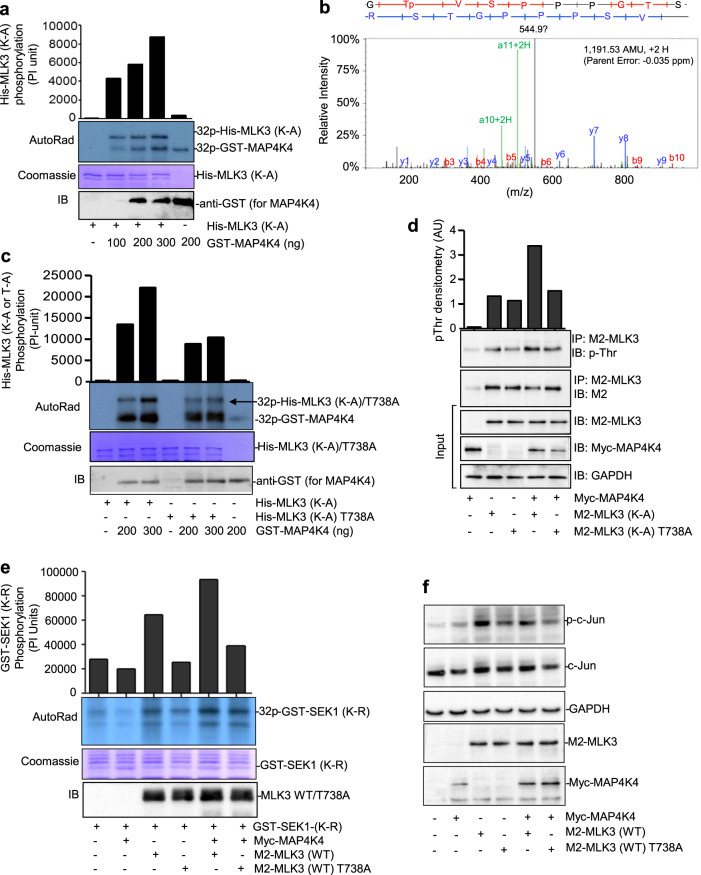
MAP4K4 directly phosphorylates MLK3 and activates its downstream signaling. **a** Bacterially expressed kinase inactive, MLK3 (K-A) protein was used in an in vitro kinase assay with increasing doses of catalytically active recombinant MAP4K4 enzyme. **b** Identification of T738 as MLK3 phosphorylation site by MAP4K4 using cold kinase assay followed by mass spectrometry. **c** The T738 as the direct phosphorylation site on MLK3 was confirmed using MLK3 (K-A) T738A mutant protein with increasing doses of MAP4K4 enzyme. **d** HEK-293 cells were transfected with mammalian expression vectors, expressing Myc-tagged MAP4K4 along with Flag-tagged MLK3 (K-A) or MLK3 (K-A) T738A. The cell extracts were used to immunoprecipitate Flag-tagged MLK3 and blotted with anti-p-Thr and anti-Flag (M2) antibodies. The total cell extracts were blotted with anti-Flag, anti-Myc tag, and GAPDH antibodies. **e** HEK-293 cells were transfected with Myc-tagged MAP4K4 along with Flag-tagged MLK3 (WT) or MLK3 (WT) T738A, respectively, and cell extracts were used to immunoprecipitate MLK3 by using anti-Flag antibody and used for kinase assays using SEK1 (K-R) as the substrate. **f** The extracts from (e) were used to blot with anti -p-c-Jun, -Jun, -Flag tag, -Myc tag, and GAPDH antibodies. The western blot and kinase assay presented; *n* = 3.

The MAP4K4 is a Ste20 member, and in yeast, Ste20 proteins are placed upstream of MAP3Ks [28, 29]. We observed that MAP4K4 is upstream of MLK3 (Fig. 1d, e) and thus, we planned to test whether MAP4K4 could directly phosphorylate MLK3. The in vitro phosphorylation assays clearly showed that purified MAP4K4 enzyme directly phosphorylates bacterially expressed, kinase-dead, MLK3 (K-A) protein (Fig. 2a) on Thr738 site as determined by Mass spectrometry (Fig. 2b). To confirm that Thr738 is a direct target of MAP4K4, a nonphosphorylable MLK3 (K-A) mutant (i.e., T738A) was created in bacterial and mammalian expression vectors. The phosphorylation of MLK3 (K-A) T738A mutant protein by MAP4K4 was significantly reduced under in vitro condition (Fig. 2c) and also in mammalian cells, as determined upon transfection with the indicated expression plasmids and blotting with p-Thr antibody (Fig. 2d). Next, we examined whether phosphorylation of T738 on MLK3 affects its kinase activity and downstream JNK activation. As shown in Fig. 2e and f, HEK-293 cells were transfected with the indicated expression vectors and used either for MLK3 kinase assay after pulldown of transfected MLK3 by FLAG-tag antibody (Fig. 2e) or blotted with a p-c-Jun antibody to determine JNK activation (Fig. 2f). The MAP4K4 could not activate the MLK3 (WT) T738A mutant’s kinase activity or its downstream c-Jun phosphorylation/activation. Whereas MLK3 (WT) was robustly activated by MAP4K4 (Fig. 2e), and that increased the downstream c-Jun phosphorylation/activation (Fig. 2f). These results together clearly suggest that MLK3 is a substrate of MAP4K4 and direct phosphorylation of MLK3 (by MAP4K4) increases its kinase activity and downstream signaling (i.e., JNK activation).

### MAP4K4 potentiate oncogenic programs in pancreatic cancer cells via MLK3 phosphorylation

**Fig. 3:**
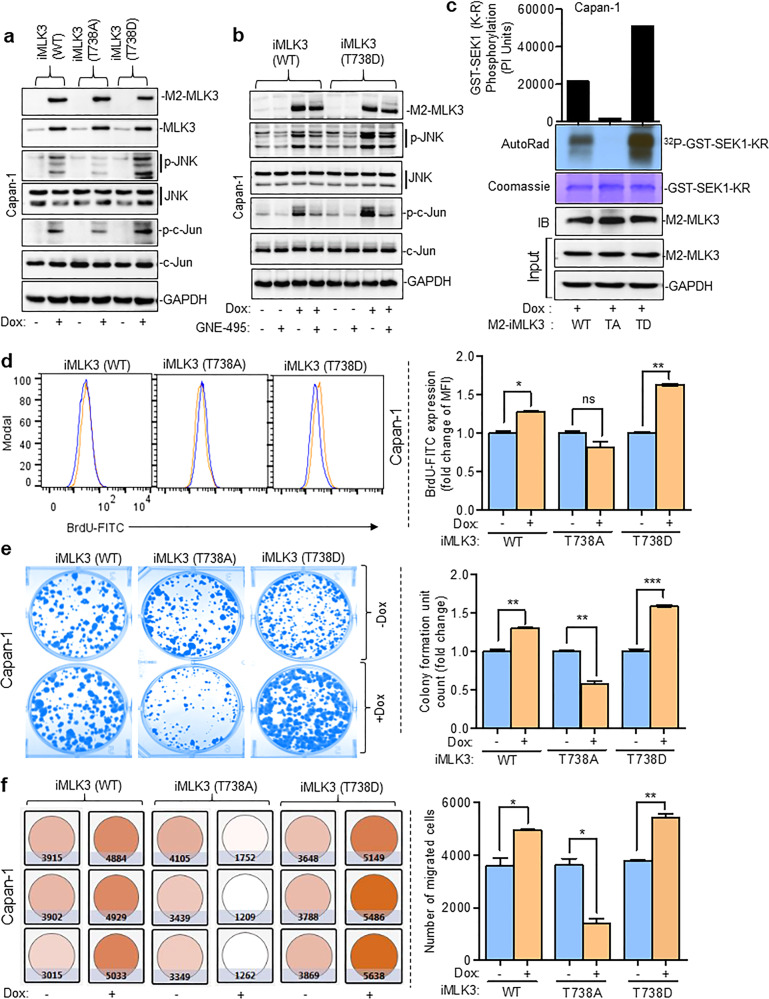
MAP4K4 phosphorylates MLK3 to promote its oncogenic potential in pancreatic cancer cells. **a** Capan-1 pancreatic cancer cells were made stable, overexpressing either doxycycline (DOX)-inducible MLK3 -WT or -T738A, or -T738D. These cells were treated with either DOX (1 µg per ml for 24 h) or vehicle, and cell extracts were blotted with anti-Flag tag (M2), -MLK3, -pJNK, -JNK, -p-c-Jun, -c-Jun, and -GAPDH antibodies. **b** Capan-1 cells, stably expressing (DOX)-inducible MLK3 -WT or -T738D, were treated with GNE-495 (2 µm for 24 h), and cell extracts were blotted with indicated antibodies**. c** MLK3 kinase activities in stable Capan-1 cell lines, expressing DOX-inducible, MLK3-WT, MLK3 (WT)-T738A, and MLK3 (WT)-T738D were determined using SEK1 (K-R) protein as the substrate. **d** Cell proliferation of stable Capan-1 cell lines, expressing DOX-inducible, MLK3-WT, MLK3 (WT)-T738A, and MLK3 (WT)-T738D was determined by flow cytometry and plotted. **e** Colony formation capacity of stable Capan-1 cell lines, expressing DOX-inducible, MLK3-WT, MLK3 (WT)-T738A, and MLK3 (WT)-T738D was determined and plotted. **f** The cell migration capacity of stable Capan-1 cell lines, expressing DOX-inducible, MLK3-WT, MLK3 (WT)-T738A, and MLK3 (WT)-T738D using the Celligo Imaging Cytometer. n = 3; quantitative data are means ± SEM. *P < 0.05, **P < 0.01, and ***P < 0.0001 (unpaired two-tailed Student’s t-test).

MAP4K4 is regarded as an important kinase regulating inflammatory pathways [5, 30] and has been reported to play roles in cancer [10]. Therefore to determine the potential role of MAP4K4 in pancreatic cancer *via* MLK3 phosphorylation, stable pancreatic cancer cell lines, Capan-1 and AsPC-1 were created, expressing either doxycycline (DOX)-inducible MLK3 -WT or -T738A (phospho-deficient) or -T738D (phosphomimetic). These stable cell lines were tested for DOX-induced MLK3 expression and downstream JNK activation (Fig. 3a and Supplementary Fig. 2a). The expression of exogenous MLK3 in these pancreatic cell lines was tightly regulated by doxycycline (Fig. 3a and Supplementary Fig. 2a), and the JNK activation was significantly higher in cells, expressing phospho-mimetic, MLK3 T738D compared to phospho-deficient MLK3 T738A (Fig. 3a and Supplementary Fig. 2a). To further confirm that activation of downstream JNK is mediated *via* MLK3 phosphorylation/activation by MAP4K4, the stable cell lines expressing inducible MLK3 -WT or -T738D were treated with MAP4K4 inhibitor GNE-495, and JNK activation was determined. The GNE-495 could not block MLK3 T738D-induced JNK and c-Jun activations (Fig. 3b), suggesting MAP4K4 is an upstream kinase that phosphorylates MLK3 to activate JNK. Since overexpressed MLK3 is constitutively active [19] and therefore it was expected that GNE-495 could only partially block JNK activation (Fig. 3b). Next, we measured the kinase activities of MLK3-WT and phosphorylation site mutants. The kinase activity of phospho-mimetic, MLK3 T738D, was significantly higher than WT or phospho-deficient mutant, MLK3 T738A (Fig. 3c and Supplementary Fig. 2b). Once the stable cell lines were characterized, we used them to determine the impact of MLK3 phosphorylation by MAP4K4 on pancreatic cancer cell proliferation (Fig. 3d and Supplementary Fig. 2c), colony formation (Fig. 3e and Supplementary Fig. 2d), and cell migration (Fig. 3f and Supplementary Fig. 2e). The proliferation of pancreatic cancer cell lines was significantly higher, expressing phosphomimetic, MLK3 T738D (Fig. 3d and Supplementary Fig. 2c). Similarly, the colony formation (Fig. 3e and Supplementary Fig. 2d) was also significantly higher in cells expressing MLK3 T738D. The pancreatic cancer cell migration was also maximal in cells expressing MLK3 T738D (Fig. 3f and Supplementary Fig. 2e). To confirm that the oncogenic function of MLK3 in part is dependent on MAP4K4-induced phosphorylation of T738 site on MLK3, we created stable cell lines expressing either inducible MLK3 -WT or -T738A, along with inducible MAP4K4. First, we determined the inducible (with DOX) expressions of MAP4K4 and MLK3 (Supplementary Fig. 3a and 3b). The induction of MLK3 T738A blocked the Capan-1 and AsPC-1 cell proliferation (Supplementary Fig. 3c and 3d). These results clearly demonstrate that MAP4K4 promotes the oncogenic potential of pancreatic cancer cell lines via phosphorylation of MLK3 on the T738 site.

### MAP4K4 is overexpressed in human pancreatic cancer tumors

**Fig. 4:**
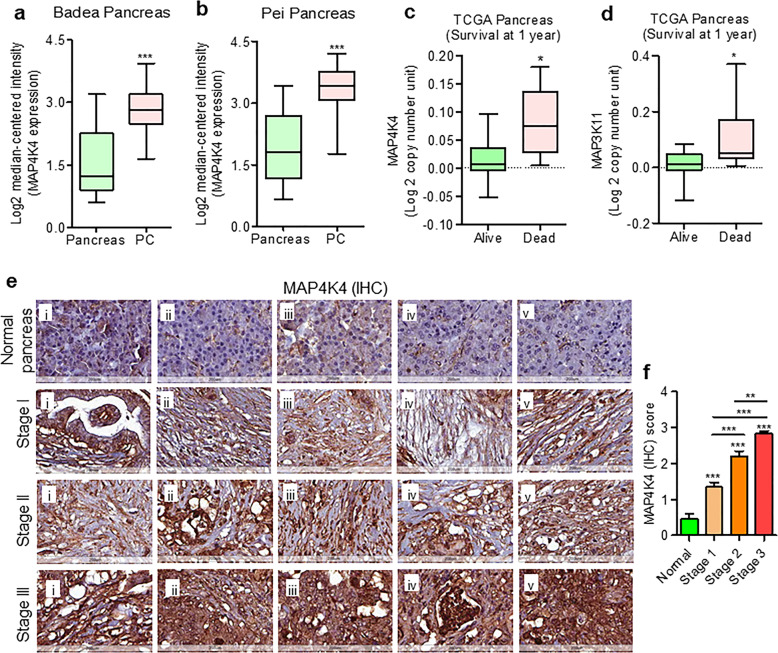
MAP4K4 expression is upregulated in human PDAC tumors. **a** and **b** The MAP4K4 mRNA expressions in normal pancreas compared to pancreatic cancer (PC) are plotted using **a** Badea Pancreas (*n* = 39) and **b** Pei Pancreas (*n* = 16 pancreas and *n* = 36 pancreatic cancer) datasets by using Oncomine software. **c** and **d**, PDAC patients’ survival and **c** MAP4K4 and **d** MLK3 (MAP3K11) expression, using TCGA data base (*n* = 13 alive and *n* = 6 dead). **e** Representative normal adjacent and PDAC tissues at different stages (I-III), stained with anti-MAP4K4 antibody. **f** The MAP4K4 staining intensities from the TMA was scored and plotted. Quantitative data are means ± SEM. *P < 0.05, **P < 0.01, and ***P < 0.0001 (unpaired two-tailed Student’s *t* test, Bonferroni’s multiple comparison test).

MAP4K4 upregulation in PDAC is reported in patients with the worst prognosis [31]. Therefore, we analyzed mRNA expression of MAP4K4 using Badea and Pei Pancreas datasets using the Oncomine platform (Thermo Fisher, Ann Arbor, MI). The MAP4K4 mRNA was significantly upregulated in pancreatic cancer tumors (Fig. 4a, b). We also analyzed the copy numbers of MAP4K4 and MLK3 (i.e., MAP3K11) using pancreas TCGA datasets and correlated with one-year survival. The upregulation of both MAP4K4 (Fig. 4c) and MLK3 (Fig. 4d) copy numbers were associated with increased mortality. To determine that the increased MAP4K4 mRNA expression corresponds to increased protein expression in pancreatic tumors, a commercially available pancreatic cancer TMA with normal and PDAC tissues at different stages of disease progression was analyzed using immunohistochemistry (IHC). Interestingly, the MAP4K4 protein was significantly upregulated in PDAC at all the stages of disease progression, compared to normal (Fig. 4e, f). However, the level of MAP4K4 expression was directly correlated with the disease’s progression (Fig. 4e, f). These results suggest that perhaps MAP4K4 might have a role in pancreatic cancer pathogenesis.

### Inhibition of MAP4K4 impedes colony formation, cell migration, cell cycle progression, and proliferation

**Fig. 5:**
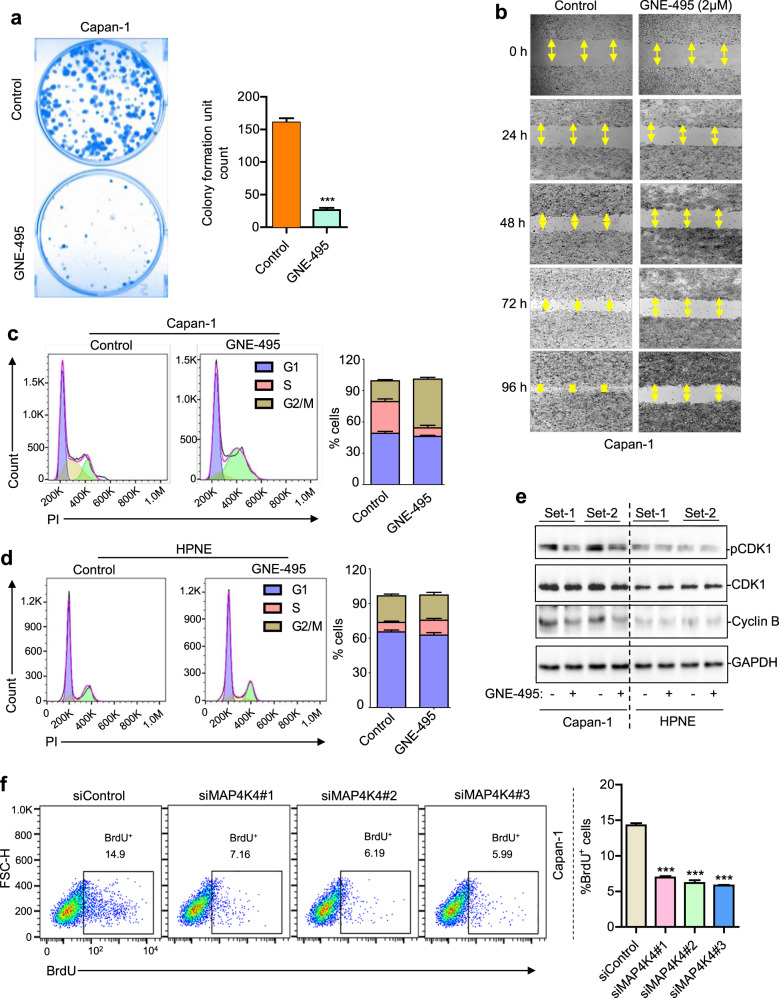
The MAP4K4 inhibition impedes colony formation, cell migration, cell cycle, and proliferation in PDAC cell lines. **a** Capan-1 cells (500 cells/well) were seeded onto a six-well plate and treated with either GNE-495 (2 µM) or vehicle for every 3rd day. Representative image shown was captured after 30 days following crystal violet staining. The colonies were counted and plotted. **b** Capan-1 cells (0.4 million/well) were seeded onto a six-well plate, and following 24 h, a scratch was made using a 200 µl micropipette tip. Cells were treated with GNE-495 (2 µM) or vehicle, and images were captured at the indicated time points using bright field microscopy. **c** and **d** the **c** Capan-1 and **d** HPNE cells were treated with GNE-495 (2 µM for 24 h.) or vehicle and stained with Propidium Iodide (PI) for cell cycle analysis by flow cytometry. **e** The Capan-1 and HPNE cells were similarly treated with GNE-495 or vehicle, and cell extracts were blotted with anti-pCDK1, CDK1, Cyclin B, and GAPDH antibodies. **f** the MAP4K4 in Capan-1 cells were knockdown using multiple siRNAs, and cell proliferation was determined by flow cytometry. Quantitative data are means ± SEM. ***P < 0.0001 (unpaired two-tailed Student’s *t* test); *n* = 3.

Several MAP4K4 small molecule inhibitors and a natural compound have been identified [26, 32–34]; however, a small molecule, GNE-495 [26], is considered more specific toward MAP4K4. To test whether GNE-495 can impede the oncogenic properties of pancreatic cancer cells, Capan-1 and PANC-1 cell lines were grown in the presence of either vehicle or GNE-495. The colony formation by PDAC cell lines was almost completely blocked by GNE-495 (Fig. 5a and Supplementary Fig. 4a). The cell migration of Capan-1 cells was also attenuated by MAP4K4 inhibitor (Fig. 5b). Next, we examined whether GNE-495 can block pancreatic cancer cell cycle progression. As shown in Fig. 5c, GNE-495 arrested pancreatic cancer cell line, Capan-1 in G2/M cell cycle stage (Fig. 5c) but not the nontumorigenic, HPNE cell line (Fig. 5d), which was also evident by decreased phosphorylation of CDK1 and expression of its associated Cyclin B in pancreatic cancer cell lines, Capan-1 (Fig. 5e) and PANC-1 (Supplementary Fig. 4b) but not in the normal pancreatic cell line, HPNE (Fig. 5e). To further confirm that the observed effect of GNE-495 is through MAP4K4 inhibition, we knocked down MAP4K4 in Capan-1 cells using specific siRNAs (Supplementary Fig. 4c) and determined cell proliferation by flow cytometry. There was a significant inhibition of cell proliferation upon knocking down MAP4K4 in Capan-1 cells (Fig. 5f). These results support that MAP4K4 inhibitor GNE-495 could potentially prevent pancreatic tumorigenesis.

### Inhibition of MAP4K4 promotes pancreatic cancer cell death

**Fig. 6:**
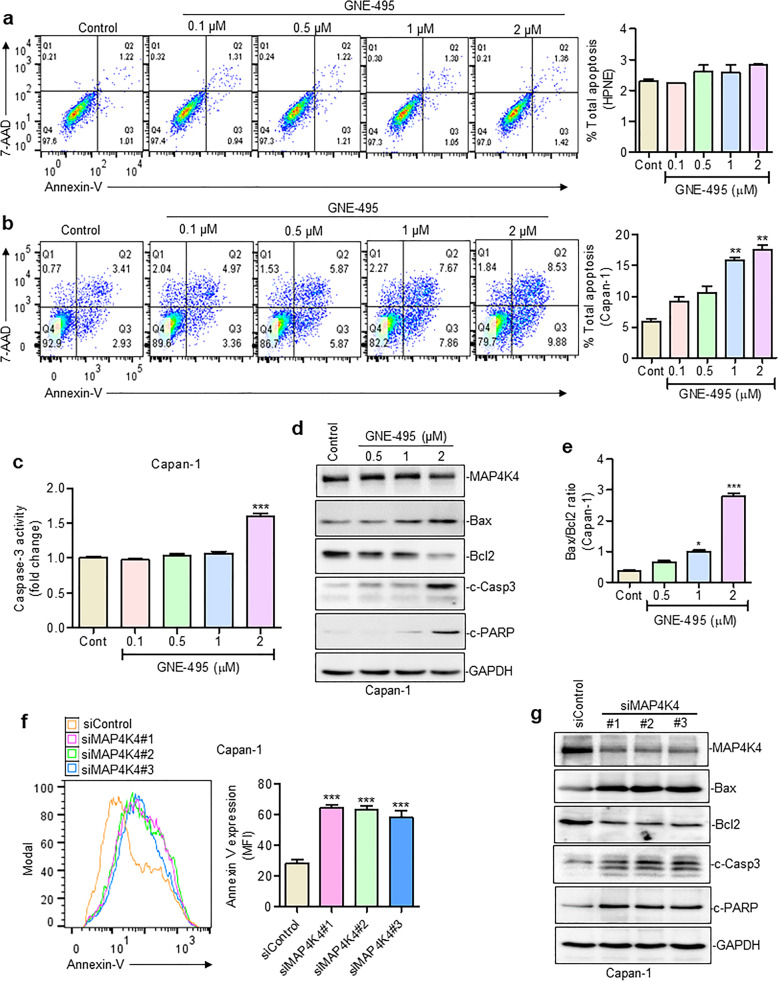
MAP4K4 inhibition promotes apoptosis in PDAC cell lines. The HPNE and Capan-1 cells were treated with GNE-495 or vehicle and stained with Annexin-V and 7-AAD to determine apoptosis: **a** HPNE and **b** Capan-1 cells by flow cytometry. **c** Capan-1 cell similarly treated like (a & b) were used for Caspase-3 activity estimation and **d** for western blotting with: anti -MAP4K4, -Bax, -Bcl2, -c-PARP, -cleaved caspase 3, and -GAPDH antibodies and **e** Bax/Bcl2 ratio was plotted. **f** the MAP4K4 was knockdown in Capan-1 using multiple siRNAs and cell death was determined 40 h-post knockdown by flow cytometry. Quantitative data are means ± SEM. *P < 0.5, **P < 0.01, ***P < 0.001. For comparisons between groups, Bonferroni’s multiple comparison test was used. The western blot and flow cytometry analyses presented; n = 3.

Since MAP4K4 inhibitor GNE-495 was able to arrest cell cycle progression of pancreatic cancer cells line (Fig. 5c, e) but not of the nontumorigenic cells (Fig. 5d), therefore we sought to determine the efficacy of GNE-495 on inducing cell death in pancreatic cells. The GNE-495 was unable to induce cell death in a nontumorigenic pancreatic cell line, HPNE (Fig. 6a); however, GNE-495 induced significant cell death in all PDAC cell lines tested in a concentration-dependent manner (Fig. 6b and Supplementary Fig. 5–c). The efficacy of GNE-495 to induce cell death in pancreatic cancer cell lines was also apparent due to its effect on increasing caspase-3 activity (Fig. 6c), Bax, cleaved -caspase 3 and -PARP expressions, and decreasing Bcl2 expression in a concentration-dependent manners (Fig. 6d, e and Supplementary Fig. 5d–e). Interestingly, the mouse PDAC cell lines, derived from a mouse model (i.e., KPC) of pancreatic cancer [35], which is quite resistant to cell death, also underwent cell death by GNE-495 in a concentration-dependent manner (Supplementary Fig. 5c, e). To rule out any non-specific effect of GNE-495 and confirm that MAP4K4 inhibition promotes cell death in pancreatic cancer cells, we knocked down MAP4K4 by using MAP4K4-specific siRNAs and determined cell death by flow cytometry and western blotting. The MAP4K4 knockdown did induce significant cell death (Fig. 6f), which was also evident by increased Bax, cleaved -Caspase 3, and -PARP expressions (Fig. 6 g). These results collectively suggest inhibition of MAP4K4 either by GNE-495 or genetic inhibition can induce cell death in PDAC cells but not in nontumorigenic cell lines.

### Pharmacological inhibitor of MAP4K4 ameliorates tumor burden and extends survival in a murine model of PDAC

**Fig. 7:**
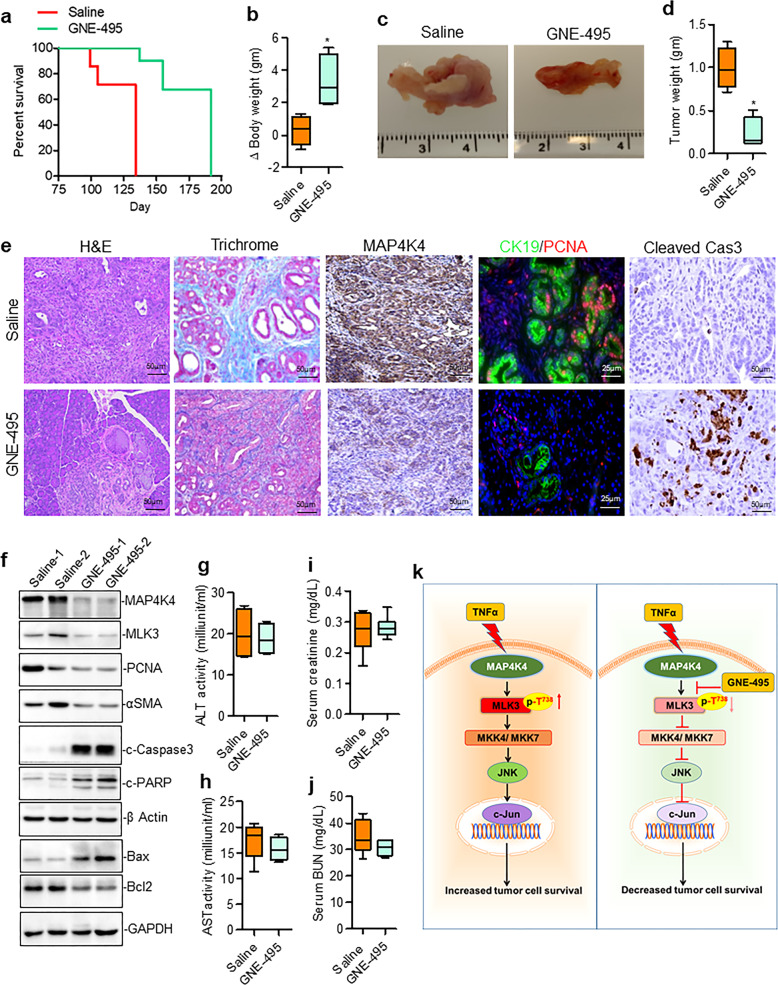
Pharmacological inhibition of MAP4K4 mitigates PDAC in vivo. The 2.5-month-old KPC mice (six mice/group) were treated either with saline or GNE-495 (3 mg/kg body weight per day) until endpoint criteria. **a** Survival in days postenrollment is displayed via Kaplan–Meier plot. **b** changes in body weight of saline and GNE-495 treated mice. **c** and **d** Whole mouse pancreas were excised from each mouse (**c**), and their weights were taken and plotted (**d**). **e** The pancreatic tissues from saline and GNE-495 treated KPC mice were stained (either IHC or IF) with H&E, Trichrome, MAP4K4, CK19/PCNA, and cleaved caspase-3, and pictures were taken. **f** KPC Mouse pancreatic tissue lysates were blotted with: anti -MAP4K4, -MLK3, -Bax, -Bcl2, -cleaved caspase 3, -c-PARP, -PCNA, -αSMA, and -GAPDH antibodies. **g** and **h** ALT and AST activities in serum of saline and GNE-495 treated KPC mice were measured and plotted. **i** and **j** Creatinine and Blood Urea Nitrogen (BUN) activities in serum of saline and GNE-495 treated KPC mice were measured and plotted. **k** Model illustrating pathway by which activation of MAP4K4 can potentiate pancreatic tumorigenesis via directly phosphorylating MLK3 on Thr738 site and activating downstream JNK activation. Conversely, GNE-495 blocks pancreatic tumorigenesis via blocking the MAP4K4-MLK3 axis and downstream signaling. Quantitative data are means ± SEM. *P < 0.05. For comparisons between two groups, Student’s two-tailed *t* test was used. For IHC, western blot and toxicity assays *n* = 6.

To assess the therapeutic relevance of GNE-495 in vivo, we created cohorts of Pdx1-Cre x LSL-KRAS^G12D^ x LSL-TP53^R172H^ (KPC) mice. These animals develop extensive PanIN disease and pancreatic adenocarcinoma, providing a reliable and consistent recapitulation of aggressive human PDAC [36]. The KPC mice were treated with either saline or GNE-495, and were maintained until endpoint criteria. The saline-treated animals’ mean survival average was 120 days, which was extended to a mean survival of 190 days in GNE-495 treated animals (Fig. 7a). The GNE-495 treated animals also maintained higher body weight than the saline-treated group (Fig. 7b). The tumor burden was measured by estimating tumor weight, which was higher in saline-treated animals (Fig. 7c, d, and Supplementary Fig. 6a). Consistent with a reduced tumor burden, GNE-495 treated mice displayed an overall reduced lesion grade as well as fewer cancerized ducts (Fig. 7e). Tissues were next evaluated by Masson’s trichrome staining (Fig. 7e), and anti-alpha Smooth Muscle Actin (α-SMA) (Supplementary Fig .6b) which indicated a substantial reduction in the tumor stroma of GNE-495-treated mice. The MAP4K4 expression in tissues was reduced significantly in mice treated with GNE-495, as also seen with the PDAC cell lines (Fig. 7e). The pancreatic ductal cell proliferation as determined by staining with CK19 (ductal marker) and PCNA or Ki-67 (proliferation markers) was visibly reduced in GNE-495 treated animals (Fig. 7e and Supplementary Fig. 6b). To determine whether these changes were associated with an increase in apoptosis, as seen in vitro, we stained tissues with activated/cleaved caspase 3, which localized to the neoplastic tissues of GNE-495-treated animals (Fig. 7e). To re-confirm our IHC/IF results with pancreatic tissues from KPC mice, the saline or GNE-495-treated pancreatic tumor extracts were blotted with antibodies for MAP4K4, MLK3, PCNA, α-SMA, cleaved caspase 3, c-PARP, Bax, and Bcl2 (Fig. 7f). The protein expression of both MAP4K4 and its downstream target MLK3 was reduced (Fig. 7f). The expression of growth marker, PCNA, and stromal marker α-SMA was also reduced in tissues from GNE-495 treated animals (Fig. 7f). The pro-apoptotic marker, cleaved caspase 3 and Bax, cleaved PARP were increased with GNE-495 treatment, whereas pro-survival Bcl2 was reduced in tumors treated with GNE-495 (Fig. 7f). The toxicity of GNE-495 was determined by estimating serum ALT, AST, creatinine and BUN, and the analyses showed no toxic effect of GNE-495 in KPC mice (Fig. 7g-j). These results suggest that GNE-495 could reduce tumor burden and extend survival of highly aggressive pancreatic cancer mouse model (i.e., KPC mouse model).

## DISCUSSION

Pancreatic cancer is associated with aberrant inflammatory cell processes, several of which appear to have driving roles in developing and maintaining the neoplastic phenotype [37]. To this end, MAP4K4 is emerging as a key mediator of the inflammatory response [5]. Classically, MAPK4K is a downstream target of the inflammatory cytokine TNFα and has been suggested to amplify TNFα-biosynthesis further, thereby enhancing inflammatory cues in a positive feedback manner [16]. However, MAP4K4 has also been shown to interact with various additional signaling networks in a highly context-specific manner [10, 38, 39]. Specifically in neuronal cells, inhibition of MAP4K4 alone [38] or along with other JNK upstream kinases [39] has been reported to paradoxically prevent cell death. However, in cancer cells, inhibition of MAP4K4 has been shown to prevent oncogenic properties [12], including cell migration [11]. While MAP4K4 is overexpressed in roughly half of PDAC patients and associates with poor clinical outcomes [31], the mechanisms through which MAP4K4 contributes to pancreatic tumorigenesis are poorly understood.

Recent evidence suggests that TNFα also activates the MAP3K family member MLK3, which is essential for downstream JNK signaling activation [18]. MLK3 has been linked to several cancers’ progression, with central roles in cell survival and proliferation [20, 40]. MLK3 is highly selective, interacting with several effectors, including the Ste20 family member Pak1 kinase, directing its activity and enhancing tumor cell proliferation [41]. Interestingly, Ste20 members are epistatically upstream of MAP3K in yeast [29, 42]. As MAP4K4 also belongs to the Ste20 family, and like MLK3 is activated downstream of TNFα and feeds into JNK signaling [16, 18], we hypothesized that MAP4K4 might act upstream of MLK3, thereby enhancing tumor development.

Our data support a functional interaction between MAP4K4 and MLK3, suggesting that MAP4K4 is an upstream activator of MLK3. For instance, MAP4K4 directly phosphorylated MLK3 on a single amino acid residue, Thr738, confirming that MLK3 is certainly a direct target of MAP4K4 and consistent with previous reports showing that MAP4K4 preferentially phosphorylates Thr residues in its substrates [15]. Moreover, our data suggest that phosphorylation of MLK3 is necessary for the tumor permissive effects of MAP4K4, as the phospho-mimetic mutant of MLK3 (T738D) was sufficient to increase all the oncogenic characteristics of pancreatic cancer cells (Fig. 3 and Supplementary Fig. 2). In contrast, the phospho-deficient MLK3 mutant (T738A) practically blocked all the oncogenic properties of MLK3 (Fig. 3 and Supplementary Fig. 2). Additionally, we found that MAP4K4 was increasingly expressed in progressive models of disease, and consistent with previous observations [31], MAP4K4 was strongly expressed in human PDAC specimens (Fig. 4). Given these results, we hypothesized that inhibition of MAP4K4 using GNE-495 might offer a potential means of a therapeutic intervention for pancreatic cancer patients. Consistent with this hypothesis, GNE-495 induced marked cell death in several PDAC cell lines (Fig. 6 and Supplementary Fig. 5). Similarly, GNE-495 extended survival in an aggressive model of murine PDAC, associated with a reduction in the tumor stroma and increased cell death in neoplastic tissues (Fig. 7d).

We, therefore, propose that MAP4K4-induced MLK3 phosphorylation is a likely means through which MAP4K4 enhances pancreatic tumorigenesis. In summary, upon activation by TNFα or other potential agonists, MAP4K4 phosphorylates MLK3 on the Thr738 site, thereby increasing activation of its oncogenic signaling effectors, such as JNK and c-Jun (Fig. 7k). As therapeutic inhibition of MAP4K4 has substantial single-agent efficacy in mice, therapeutic strategies targeting MAP4K4 warrant continued exploration in PDAC, potentially offering a new therapeutic approach for a disease where there is currently no effective treatment.

## Materials and Methods

### Cell lines and treatments

The human pancreatic cancer cell lines, Capan-1, PANC-1, BxPC-3, AsPC-1, and human embryonic kidney 293 (HEK-293) cells were purchased from American Type Culture Collection (ATCC, Manassas, VA), and HEK-293-FT cell line was procured from Life Technologies, USA. The murine PDAC cell line, KPC-105 was obtained from our collaborator (Paul Grippo, UIC, Chicago). Capan-1 cells were cultured in Iscove’s Modified Dulbecco’s Medium (IMDM) medium, supplemented with 10% fetal bovine serum (FBS), 1% L-glutamine, and 100 units/ml penicillin/streptomycin. PANC-1, BxPC-3, HEK-293, and HEK-293-FT cell lines were cultured in DMEM medium, supplemented with 10% fetal bovine serum (FBS), 1% L-glutamine, and 100 units/ml penicillin/streptomycin. AsPC-1 cells were maintained in RPMI-1640 medium supplemented with 10% fetal bovine serum (FBS), 1% L-glutamine, and 100 units/ml penicillin/streptomycin. The GNE-495 and CEP-1347 was initially provided by Dr. Subhash C. Sinha (Weill Cornell Medicine, New York, USA) and later CEP-1347 was procured from Tocris, USA (catalog no. 4924). These inhibitors were solubilized in DMSO at a concentration of 50 mM for GNE-495 and 4 mM for CEP-1347 (stock solution) and stored at −20 °C in small aliquots in amber glass vials.

### Expression vectors, siRNAs and mutagenesis

Human MAP4K4 cDNA was PCR amplified from pDONR223-MAP4K4 (Addgene # 23486) using primers hMAP4K4-EcoRI-F: CGGAATTCATGGCGAACGACTCCCCTGCAAAAAGTCTG and hMAP4K4-XhoI-R CCGCTCGAGCCAGCTCAGAAGAGAAGTCCTGCC and cloned into pGEM-T vector. Cloned MAP4K4 cDNA was then retrieved from the pGEM-T and sub-cloned into the EcoRI and XhoI sites in pcDNA3.1-Myc vector. Correct insertion was confirmed by colony PCR, restriction enzyme digestion, and sequencing. The pRK5-Flag-MLK3 and kinase-deficient pRK5-Flag-MLK3 (K-A) mammalian expression vectors are described earlier [19]. The GST versions of MLK3 mutants were generated by PCR using pRK5-Flag-MLK3 wild type or kinase deficient as template and sub-cloned into bacterial expression vector (pGEX4T). The mammalian and bacterial expression vectors of mutant MLK3 were generated using the QuikChange Lightning Site-Directed mutagenesis kit (Agilent Technologies, USA. catalog no. 200518) and sequenced using Sanger’s method at UIC-RRC facility. For inducible lentiviral vector construction, the human full-length M2-MLK3 WT or M2-MLK3 T738 mutants coding sequences (CDS) were cloned into the pDONOR221 vector using BP Clonase (Life Technologies, USA, catalog no: 11789100), attB1: GGGGACAAGTTTGTACAAAAAAGCAGGCTAAAATGGCGAACGACTCCCCTGCAAAAAGTCTGGTGGACATCG and attB2: GGGGACCACTTTGTACAAGAAAGCTGGGTCTATCACAGATCCTCTTCTGAGATGAGTTTTTGTTCG primers. The LR recombination reaction was performed with the entry vector using LR Clonase (Life Technologies, USA. catalog no.11791020) to generate lentiviral vector in the pLIX_403 backbone. The validated sets of MLK3 and MAP4K4, and scrambled (control) siRNAs (Supplementary Table 1) were purchased from Dharmacon (Horizon Discovery).

### Virus production and transduction

Inducible lentiviruses were produced by co-transfecting the lentiviral vectors for MLK3 WT, MLK3 T738 mutants, with psPAX2, and pMD2G packaging plasmids into HEK 293FT cells (Life Technologies, USA) using Lipofectamine 2000 (Life Technologies, USA. catalog number: 11668019). To achieve a MOI of 0.3–0.5, titrations were performed and the infection efficacy of was determined using Western blotting. The Capan-1 and AsPC-1 cells were infected with an optimal volume of virus in a medium containing 5 µg/ml polybrene (Sigma-Aldrich, USA, catalog no. TR-1003). Stable cell lines were selected using 3–5 µg/ml Puromycin (GoldBio, USA, catalog no: P-600–100) for 10 days.

### Recombinant protein expression, immunoprecipitation, immunoblotting, kinase assay, and phosphorylation site determination

For in vitro assays, His-MLK3 (K-A), His-MLK3 (K-A) T738A, GST-SEK1 (K-R)/MKK4 (K-R), were expressed in bacteria (strain BL21; Pharmacia, NJ) and purified over His resin and GSH beads. Proteins were eluted, dialyzed, and stored at −20 °C in 50% glycerol. HEK-293 cells were lysed in NP-40 lysis buffer and cell lysates were prepared and immunoblotting was performed using a standard method as described previously [18, 19]. The recombinant MAP4K4 and MLK3 from HEK-293 cell extracts were immunoprecipitated using protein A-sepharose preabsorbed with either anti-Flag (M2) or anti-Myc (9E10) antibody or anti-c-Myc or anti-Flag (M2) -magnetic beads. The proteins were separated on denaturing SDS/PAGE and transferred on to PVDF membrane and blotted with antibodies as indicated. Kinase assay was performed as described earlier [19, 21] in presence of either His-MLK3 (K-A) or His-MLK3 (K-A) T738A or GST-SEK1 (K-R)/MKK4 (K-R) as the substrates. For in vitro kinase assays, bacterially expressed MLK3 protein was incubated with MAP4K4 active enzyme, prior to the assay. The phosphorylation sites of MLK3 was determined upon incubating bacterially expressed His-MLK3 (K-A) protein with active MAP4K4 enzyme. The phosphorylated MLK3 protein was excised and sent out (UAMS Proteomics Core Lab, Little Rock, AR) to determine the p-sites by Mass Spectrometry.

### Cell cycle and cell apoptosis analysis

For the cell cycle assay, Control and GNE-495 (2 µM) treated cells were harvested at 24 h. Pancreatic cancer cell line were washed in ice cold 1X PBS and fixed in chilled 70% ethanol (200 proof) while vortexing and stored at −20 °C. Further, cells were washed using cold FCM buffer twice and incubated with RNase (100 µg/ml) for 5 min. Furthermore, cells were stained with Propidium Iodide (50 µg/ml) for 30 min on ice. For cell death and apoptosis analysis, control and GNE-495 (2 µM) treated cells stained with the Annexin V-FITC/7AAD kit (BioLegend, catalog no. 640930) according to the manufacturer’s instructions. Data was analyzed flow cytometry (LSR Fortessa, BD) using Flow Jo v10 software.

### Caspase-3 activity

The caspase-3 activity in cell lysates of Capan-1 cells were determined as earlier described method [43]. Briefly, whole-cell extract were prepared from control and GNE-495 treated (24 h) Capan-1 cells using NP-40 lysis buffer. The DEVD-AFC (Enzo Life Sciences, USA) was used as a substrate for caspase-3 activity. Fluorescence was read at excitation 400/30 nm and emission 508/20 nm using a plate reader (BioTek Microplate Readers, USA). The fluorescence obtained for caspase-3 was normalized by their respective protein concentrations.

### In vivo pancreatic cancer (KPC) model

The Pdx1-Cre x LSL-KRAS^G12D^ x LSL-TP53^R172H^ (KPC), mice were bred as the method described earlier [36]. All animal experiments were performed under a protocol approved by UIC-IACUC. The KPC (6 mice/group) mice (age 2.5 months, both sex) were administered intraperitoneally with vehicle control or GNE-495 (3 mg/Kg/BW), daily up to 3 months. The KPC mice were sacrificed when moribund or showing clear signs of health decline, e.g., fur loss, weight loss, or lethargy. The serum was used for toxicity assays and organs were used for Western blots and IHC analyses.

### Statistical analysis

To compare the two groups, data were analyzed by unpaired two-tailed student’s *t* test. For comparison of multiple groups, data were analyzed by one-way ANOVA followed by Bonferroni’s multiple comparison test using the GraphPad Prism (Version 5). The differences were considered significant when p values were <0.05. Unless otherwise indicated, all the experiments presented in the manuscript were repeated a minimum of two times with a minimum of three replicates.

## Supplementary information

Supplemental Information
