# Supplementary material for: MAP4K4 promotes pancreatic tumorigenesis via phosphorylation and activation of mixed lineage kinase 3
Source: Oncogene. 2021 Sep 13;40(43):6153–65. doi: 10.1038/s41388-021-02007-w (PMC8553609; doi:10.1038/s41388-021-02007-w)
Supplement: Supplementary file 1 — Supplemental Information [file 41388_2021_2007_MOESM1_ESM.pdf]

Supplementary Information

MAP4K4 promotes pancreatic tumorigenesis via phosphorylation and activation of MLK3

Sunil Kumar Singh, Sandeep Kumar, Navin Viswakarma, Daniel R. Principe, Subhasis Das, Gautam Sondarva, Rakesh Sathish Nair, Piush Srivastava, Subhash C. Sinha, Paul J Grippo, Gregory R.J. Thatcher, Basabi Rana, and Ajay Rana[#]

Supplementary methods, table and figures

Western blot analysis, Antibodies and reagents. Equal amounts of total cell extracts or immunoprecipitated samples were fractionated by SDS-PAGE, transferred to PVDF membranes, and subjected to Western blot analysis utilizing various antibodies. Antibodies and dilutions were as follows: anti-p-c-Jun, 1:1000 (clone D47G9; Cell Signaling Technology; cat# 3270); anti-c-Jun, 1:2000 (clone 60A8; Cell Signaling Technology; cat# 9165); anti-p-ERK1/2, 1:2000 (clone D13.14.4E; Cell Signaling Technology; cat# 4370); anti-ERK1/2, 1:2000 (clone 137F5; Cell Signaling Technology; cat# 4695); anti-p-P38, 1:2000 (clone D3F9; Cell Signaling Technology; cat# 4511); anti-P38, 1:2000 (Cell Signaling Technology; cat# 9212); anti-p-CDK1, 1:1000 (Tyr15; Cell Signaling Technology; cat# 9111); anti-CDK1, 1:1000 (Cell Signaling Technology; cat# 77055); anti-Cyclin B, 1:1000 (clone V152, Cell Signaling Technology; cat# 4135); anti-c-caspase3, 1:1000 (clone Asp175; Cell Signaling Technology; cat# 9661); anti-c-PARP, 1:1000 (clone Asp214; Cell Signaling Technology; cat# 9541) anti-Myc-Tag, 1:1000 (Cell Signaling Technology; cat# 2272); anti-MLK3, 1:3000 (clone EP1460Y; Abcam; cat# ab51068); anti-αSMA, 1:2000 (Abcam; cat# ab5694); anti-MAP4K4, 1:3000 (Proteintech; cat# 55247-1-AP); anti-GAPDH, 1:5000 (Proteintech; cat# 10494-1-AP); anti-M2, 1:2000 (Sigma; Cat# F1804); anti-pJNK, 1:5000 (Promega; Cat# V7932); anti-JNK, 1:500 (clone D-2; Santa Cruz Biotechnology; Cat# sc-7345), anti-pThr, 1:500 (clone H-2; Santa Cruz Biotechnology; Cat# sc-5267); anti-Bax, 1:500 (clone N-20; Santa Cruz Biotechnology; Cat# sc-493); anti-Bcl2, 1:500 (clone C-2; Santa Cruz Biotechnology; Cat# sc-7382); anti-PCNA, 1:500 (clone PC10; Santa Cruz Biotechnology; Cat# sc-56); anti-CK19, 1:100 (clone TROMA-3; Millipore Sigma; Cat# MABT913). Species specific secondary horseradish peroxidase (HRP)-coupled antibodies (Jackson ImmunoResearch; Peroxidase AffiniPure Goat Anti-Rabbit IgG (H+L) cat# 111-035-045 and Rabbit Anti-Mouse IgG (H+L) cat# 315-035-045) were used. Protein G-Sepharose (cat# GE17-0618-01) and Glutathione Sepharose® 4B (cat# GE17-0756-01) coated beads were purchased from GE Healthcare. All chemicals or reagents including anti-Flag-M2 (cat# M8823) magnetic beads were from Sigma-Aldrich, USA. GST-MAP4K4 active enzyme was

obtained from SignalChem (cat# M26-11G), Canada and Prolong-diamond Antifade mount with DAPI (cat# P36962) was from Life technologies, USA.

Immunohistochemistry, immunostaining and microscopy. Human tissue microarrays (TMAs) were purchased from US Biomax (cat# PA1001). One hundred cores of pancreatic normal and carcinoma tissues were used for this study. Each TMA had a 5-µm-thickness and 1mm core diameter of independent diseased or normal tissue spot from a specimen that was selected and pathologically confirmed. Formalin-fixed and paraffin-embedded pancreatic cancer tumors of KPC mice were processed and prepared for tissue sectioning, using standard protocol for immunohistochemistry (IHC), paraffin-embedded tissue sections were deparaffinized in xylene, rehydrated through 100% ethanol to TBST wash solution. The antigenic epitope was retrieved using sodium citrate buffer (pH 6.0) in decloaking chamber at 95^0C for 20 mins. Peroxidase quenching was performed using BLOXALL (Vector Lab, USA, cat# SP-6000-100) and blocked in 5% goat serum for 30 min. Slides were then incubated with primary antibodies against MAP4K4 (dilution, 1:200), cleaved-Caspase3 (dilution, 1:100), in 1X antibody diluent (DAKO, USA) for overnight at 4^0C in humidified chamber and washed three times with TBST. Slides were incubated with respective secondary-HRP antibodies (Vector Lab, USA) and developed using DAB (Vector Lab, USA, cat# SK-4105). The nuclei were counterstained with Hematoxylin (RICCA, USA, cat# R3530000) for 5 min, rinsed in deionized water and mounted with Krystalon mounting medium (Millipore Sigma, USA, cat# 64969). Bright field microscopy was done using NIS-Elements imaging software in Nikon Eclipse Ti Microscope. For immunofluorescence (IF) analysis, Capan-1 cells were grown on the cover glass, and after 48 h cells were fixed in 4% paraformaldehyde and permeabilized using permeabilization buffer. Further, cells were incubated with anti-MAP4K4 and MLK3 antibodies, overnight at 4^0C. After washing with 1X PBS, slides were incubated with Alexa Flour 549-labeled anti-rabbit IgG (Abcam 1:1000) for MAP4K4 and Alexa Flour 488-labeled anti-mouse IgG (Abcam, 1:1000) for MLK3 for 45 min at room temperature. After washing with 1XPBS, the nuclei were stained with DAPI using ‘prolong-diamond Antifade mount with DAPI’ (Life technologies, USA. Cat# P36962) and pictures taken using

confocal microscope (Zeiss LSM microscope 710, Carl Zeiss, Inc.) equipped with Zeiss confocal scanning optics.

Colony formation assay. Briefly, Capan-1 and PANC-1 cells (500 cells/well) were seeded onto six well plate and treated with 2 µM GNE-495 daily up to three days, and then cells were treated with 2 µM GNE-495, on every 3rd day. Also, AsPC-1-iMLK3 (WT, T738A and T738D) and Capan-1-iMLK3 (WT, T738A and T738D) cell lines were seeded onto six well plate and treated with vehicle or Dox for alternate day. On day 21, Colonies were washed with 1X PBS, fixed with 4% paraformaldehyde and stained with 0.1% crystal violet for imaging. Images were captured using a bright field microscope (Nikon Eclipse Ti Microscope).

Cells migration assays. To determine effect of GNE-495 on cellular migration, Capan-1 cells (0.4 X 10^6 cells/well) were seeded on to a six-well plate and treated with GNE-495 (2 µM) or vehicle. The images of specific wound areas were captured by phase contrast microscopy (Nikon Eclipse Ti inverted microscope) at 0, 24, 48, 72 and 96 hrs. The width of the wounds was measured using the NIS-Element software associated with the microscope. Capan-1-iMLK3 (WT, T738A and T738D) and AsPC-1-iMLK3 (WT, T738A and T738D) cell lines (30,000 cells/well) were plated in IMDM and RPMI media containing 10% FBS on Oris™ Cell Migration plates (Platypus, NJ, USA) containing cell seeding stoppers. The cells were allowed to attach and spread for 18h prior to manual removal of the stoppers. The cells were washed and media replaced with growth media containing 10% FBS and Doxycycline. A circular cell-free zone, the migration area, was defined. In the operational version of the assay cellular confluency determined using the stain free bright field method. Mean intensity measurements were used to gate out cells with dim signals from debris. Total cellular area (%) in the migration zone were used to calculate percent increase in migration (at 48h) or % wound healing. The plate reading done using the Celligo Imaging Cytometer (Nexcelcom BioSciences, MA, USA).

Toxicity Assays. For MAP4K4 inhibitor toxicity assessment, we determined Alanine Aminotransferase (ALT), Aspartate Aminotransferase (AST), Creatinine and Blood Urea

Nitrogen (BUN) in serum of Saline and GNE-495 treated mice using commercially available kits from Sigma, catalog no. MAK052 for ALT and MAK055 for AST, Crystal Chem, catalog no. 80350, Thermo Fisher Scientific, catalog no. EIABUN respectively.

Supplementary Table S1. MAP3K11 (i.e. MLK3) and MAP4K4 siRNAs Sequences

siRNA number	Gene	Sequences
#1	MAP3K11	CAUGGUACCUGGAUUCAGA
#2	MAP3K11	UGGCGUAGCUGUUAACAAG
#1	MAP4K4	GGGAAGGUCUAUCCUCUUA
#2	MAP4K4	GACCAACUCUGGCUUGUUA
#3	MAP4K4	UAAGUUACGUGUCUACUAU
#4	MAP4K4	UAUAAGGGUCGACAUGUUA

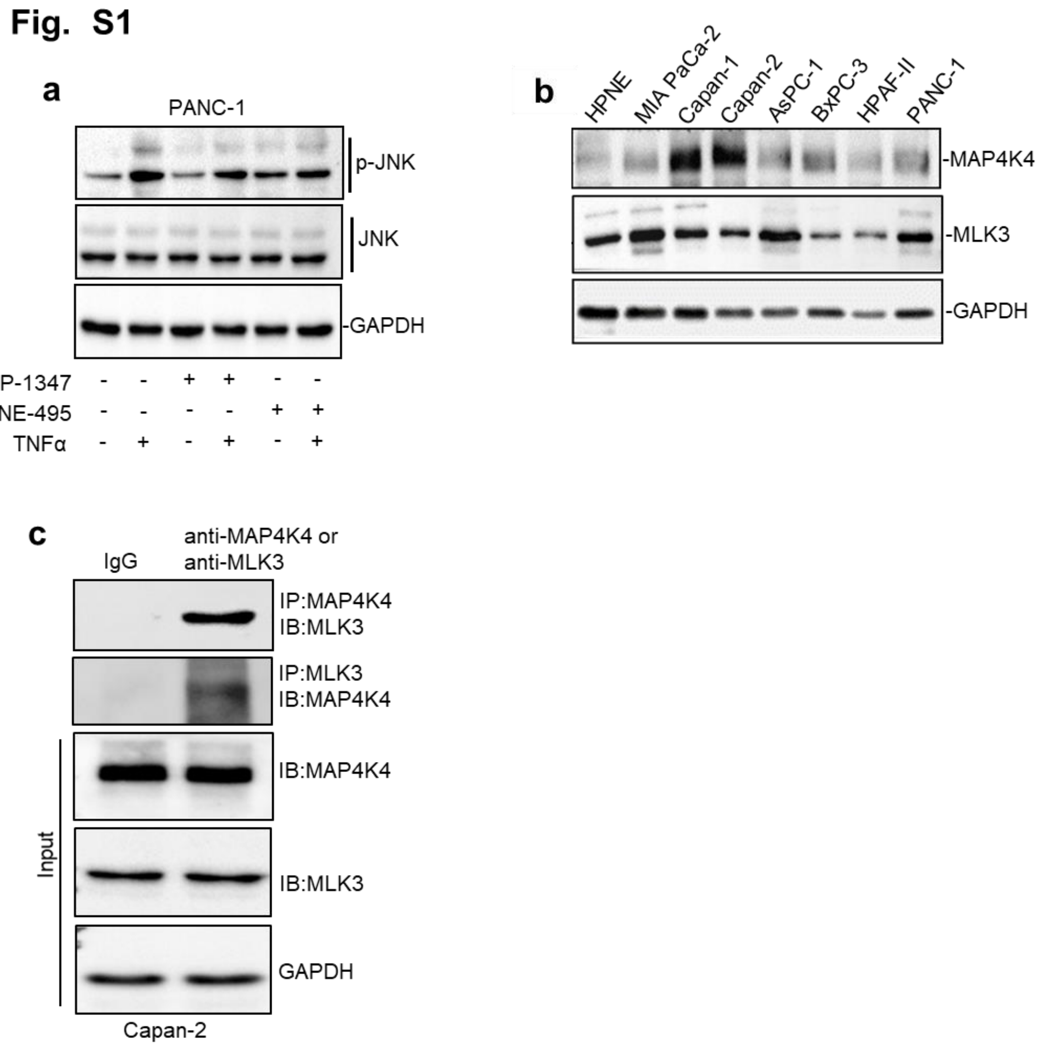

Supplementary Fig. 1 MAP4K4 associates with MLK3 and regulates downstream signaling in PDAC. **(a)** PANC-1 cells were pretreated either with MAP4K4 inhibitor, GNE-495 (2 µM) or MLKs inhibitor, CEP-1347 (500 nM) for 24 hrs. in presence or absence of TNFα (5 ng for 15 min), and cell extracts were blotted with: anti- p-JNK, JNK, and GAPDH antibodies. **(b)** Cell extracts of immortalized normal human pancreatic cell line (HPNE) and indicated pancreatic cancer cell lines were blotted with anti -MAP4K4, -MLK3, and -GAPDH antibodies. **(c)** Endogenous MAP4K4 or MLK3 from Capan-2 cells were immunoprecipitated and blotted either with anti -MLK3 or -MAP4K4 antibodies to determine their interaction.

Fig. S2

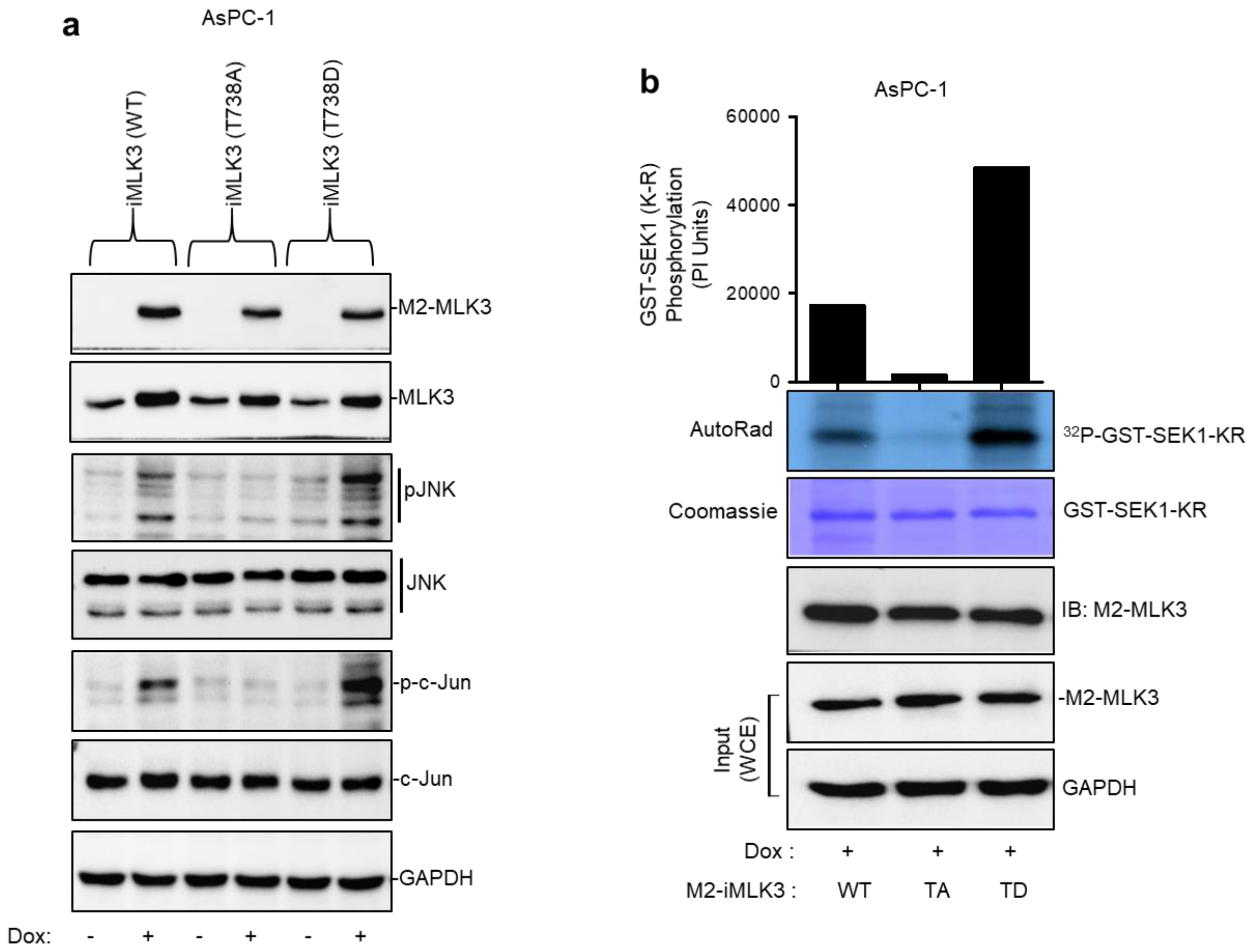
a
AsPC-1
iMLK3 (WT)
iMLK3 (T738A)
iMLK3 (T738D)
M2-MLK3
MLK3
pJNK
JNK
p-c-Jun
c-Jun
GAPDH
Dox: - + - + - +
b
AsPC-1
GST-SEK1 (K-R) Phosphorylation (PI Units)
60000
40000
20000
0
AutoRad
32P-GST-SEK1-KR
Coomassie
GST-SEK1-KR
IB: M2-MLK3
M2-MLK3
Input (WCE)
GAPDH
Dox : + + +
M2-iMLK3 : WT TA TD

Fig. S2 continue…

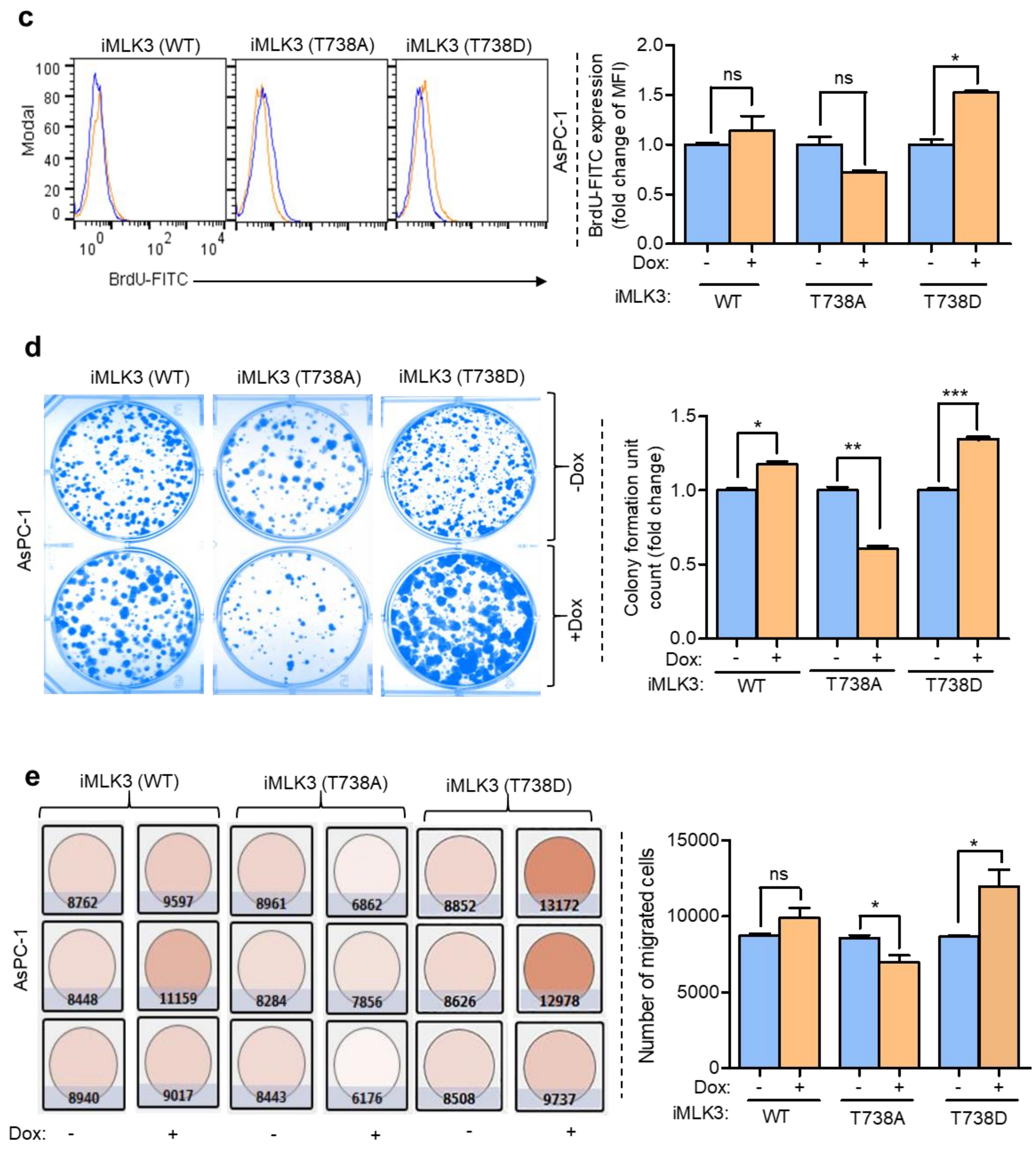

Supplementary Fig. 2 MAP4K4 phosphorylates MLK3 and potentiate oncogenic properties of pancreatic cancer cells. **(a)** Stable AsPC-1 pancreatic cancer cell lines, overexpressing either doxycycline (DOX)-inducible MLK3 -WT or -T738A, or -T738D were made. These cell lines were treated with either DOX (1µg per ml for 24hrs.) or vehicle, and cell extracts were blotted with anti -Flag tag (M2), -MLK3, -pJNK, -JNK, -p-c-Jun, -c-Jun and -GAPDH antibodies. **(b)** MLK3 kinase activities in stable AsPC-1 cell lines, expressing DOX-inducible, MLK3-WT, MLK3 (WT)-T738A, and MLK3 (WT)-T738D were determined using SEK1 (K-R) protein as the substrate. **(c)** Cell proliferation of stable AsPC-1 cell lines, expressing DOX-inducible, MLK3-WT, MLK3 (WT)-T738A, and MLK3 (WT)-T738D was determined by flow cytometry and plotted. **(d)** Colony formation capacity of stable AsPC-1 cell lines, expressing DOX-inducible, MLK3-WT, MLK3 (WT)-T738A, and MLK3 (WT)-T738D was determined and plotted. **(e)** The cell migration capacity of stable AsPC-1 cell lines, expressing DOX-inducible, MLK3-WT, MLK3 (WT)-T738A, and MLK3 (WT)-T738D using the Celligo Imaging Cytometer. n = 3; quantitative data are means ± SEM. *P < 0.05, **P < 0.01, and ***P < 0.0001 (unpaired two-tailed Student's t-test).

Fig. S3

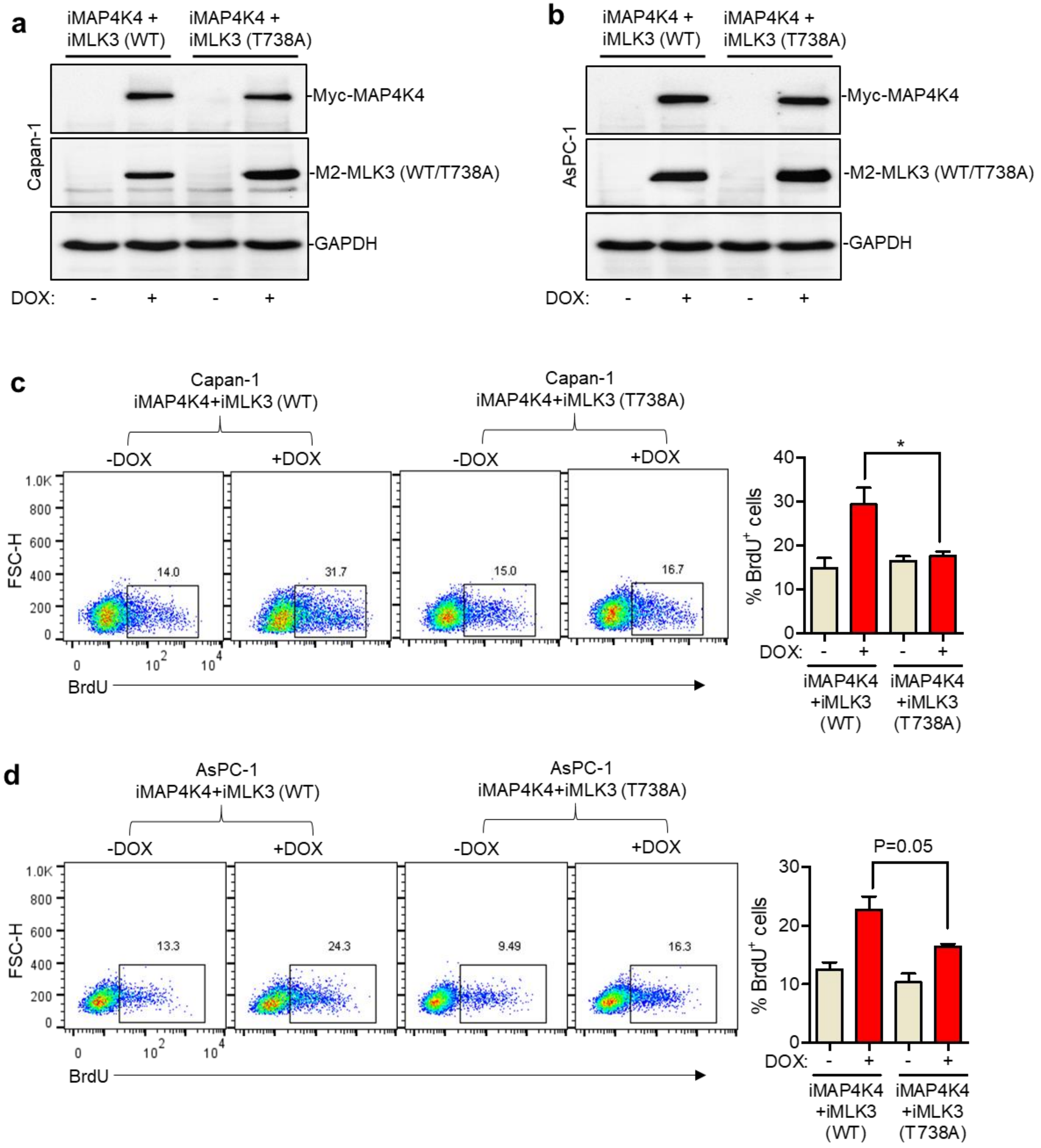
a
iMAP4K4 + iMLK3 (WT)
iMAP4K4 + iMLK3 (T738A)
Capan-1
Myc-MAP4K4
M2-MLK3 (WT/T738A)
GAPDH
DOX: - + - +
b
iMAP4K4 + iMLK3 (WT)
iMAP4K4 + iMLK3 (T738A)
AsPC-1
Myc-MAP4K4
M2-MLK3 (WT/T738A)
GAPDH
DOX: - + - +
c
Capan-1 iMAP4K4+iMLK3 (WT)
Capan-1 iMAP4K4+iMLK3 (T738A)
-DOX
+DOX
-DOX
+DOX
FSC-H
BrdU
14.0
31.7
15.0
16.7
% BrdU+ cells
*
DOX: - + - +
iMAP4K4 +iMLK3 (WT)
iMAP4K4 +iMLK3 (T738A)
d
AsPC-1 iMAP4K4+iMLK3 (WT)
AsPC-1 iMAP4K4+iMLK3 (T738A)
-DOX
+DOX
-DOX
+DOX
FSC-H
BrdU
13.3
24.3
9.49
16.3
% BrdU+ cells
P=0.05
DOX: - + - +
iMAP4K4 +iMLK3 (WT)
iMAP4K4 +iMLK3 (T738A)

Supplementary Fig. 3 The phosphorylation of MLK3 by MAP4K4 on T738 is necessary for pancreatic cancer cell proliferation. **(a)** Capan-1 and **(b)** AsPC-1 cells were made stable expressing doxycycline (DOX)-inducible MLK3 –WT or -T738A (phosphodeficient) mutant along with DOX-inducible MAP4K4. The expression of Myc-tagged MAP4K4 and Flag-(M2)-tagged MLK3 was determined upon DOX induction for 24 hrs. **(c** and **d)** The cell proliferation of stable (c) Capan1 and (d) AsPC-1 cells was determined in the presence or absence of DOX and plotted. n = 3; quantitative data are means ± SEM. *P < 0.05 (unpaired two-tailed Student's t-test).

Fig. S4

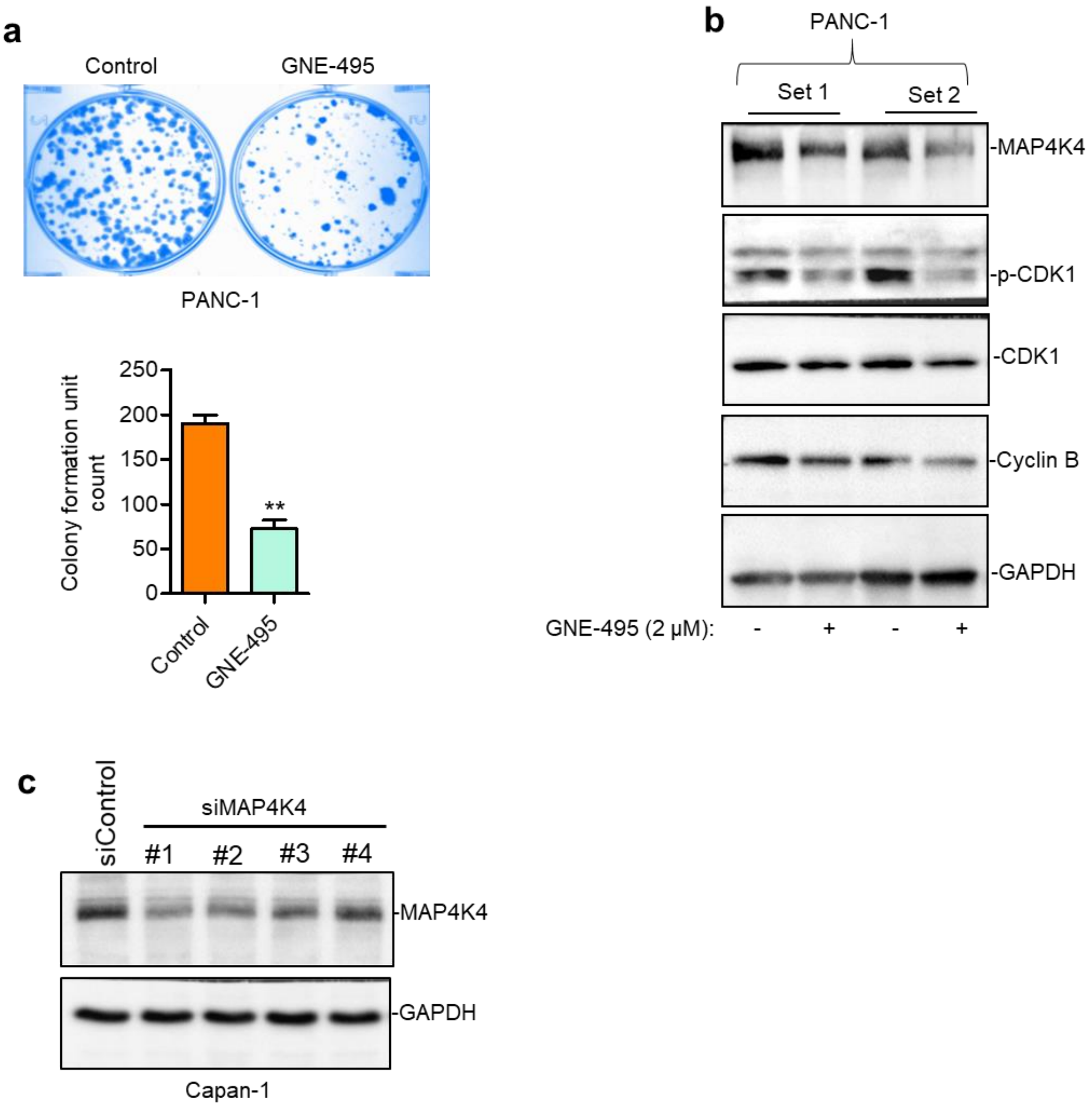

Supplementary Fig. 4 The MAP4K4 inhibition impedes colony formation and cell cycle progression in the PDAC cell line. **(a)** PANC-1 cells (500 cells/well) were seeded onto a six-well plate and treated with either GNE-495 (2 μM) or vehicle every 3rd day. Representative image shown was captured after 30 days following crystal violet staining. The colonies were counted and plotted. **(b)** The PANC-1 cells were similarly treated with GNE-495 (2 μM) or vehicle, and cell extracts were blotted with anti -pCDK1, -CDK1, -Cyclin B, and -GAPDH antibodies. **(c)** The MAP4K4 was knockdown in the Capan-1 cell line using 4 different siRNAs and protein expression determined by western blotting.

Fig. S5

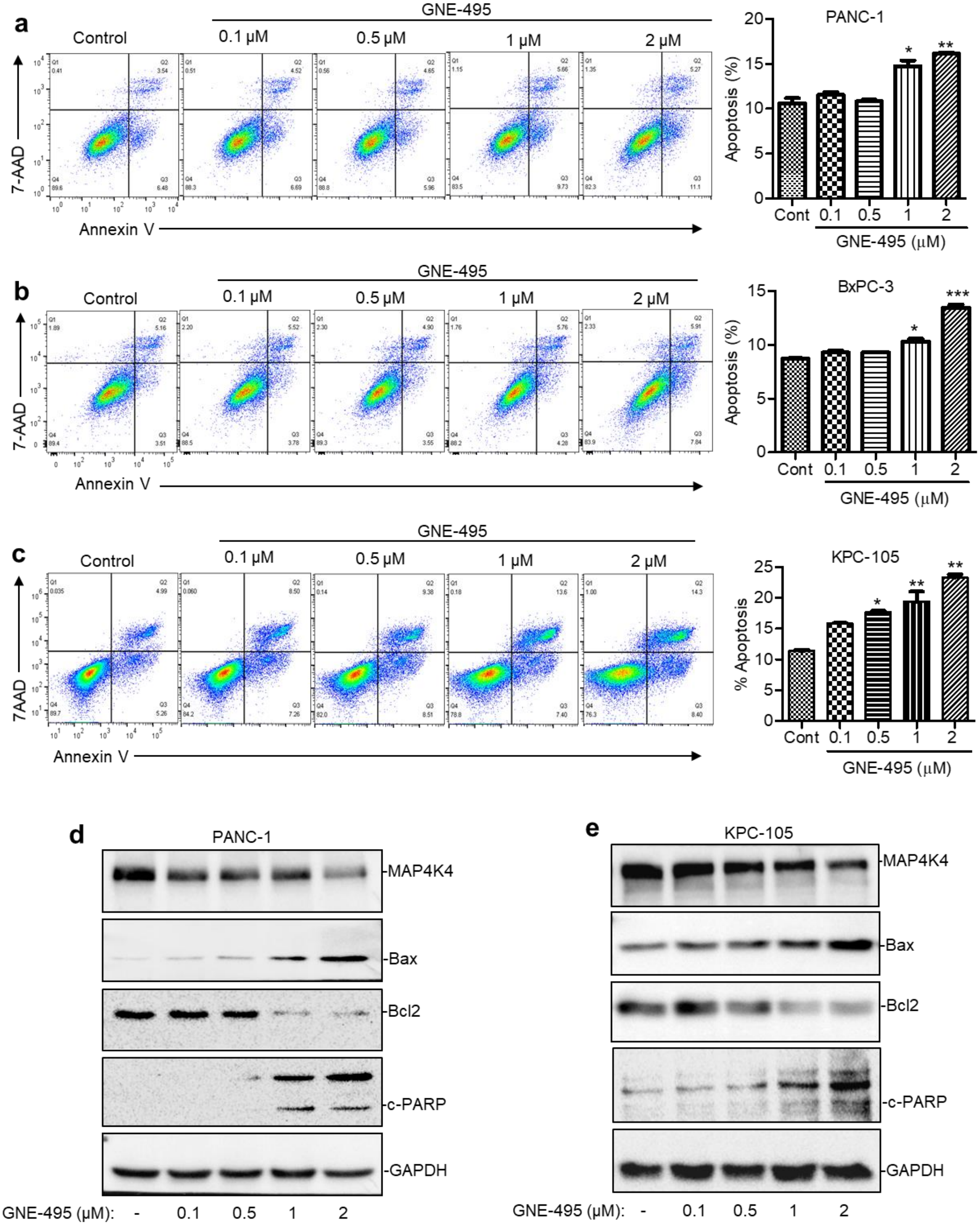
a
GNE-495
Control
0.1 μM
0.5 μM
1 μM
2 μM
7-AAD
Annexin V
PANC-1
Apoptosis (%)
Cont
0.1
0.5
1
2
GNE-495 (μM)
b
GNE-495
Control
0.1 μM
0.5 μM
1 μM
2 μM
7-AAD
Annexin V
BxPC-3
Apoptosis (%)
Cont
0.1
0.5
1
2
GNE-495 (μM)
c
GNE-495
Control
0.1 μM
0.5 μM
1 μM
2 μM
7AAD
Annexin V
KPC-105
% Apoptosis
Cont
0.1
0.5
1
2
GNE-495 (μM)
d
PANC-1
MAP4K4
Bax
Bcl2
c-PARP
GAPDH
GNE-495 (μM): - 0.1 0.5 1 2
e
KPC-105
MAP4K4
Bax
Bcl2
c-PARP
GAPDH
GNE-495 (μM): - 0.1 0.5 1 2

Supplementary Fig. 5 MAP4K4 inhibition promotes apoptosis in PDAC cell lines. The PANC-1, BxPC-3, and KPC-105 cells were treated with GNE-495 or vehicle and either stained with Annexin-V and 7-AAD to determine apoptosis by flow cytometry: **(a)** PANC-1 **(b)** BxPC-3 and **(c)** KPC-105 cell lines. **(d** and **e),** PANC-1 and KPC-105 cells were similarly treated like (**a** and **c**) and used for western blotting with: anti -MAP4K4, -Bax, -Bcl2, -c-PARP, and -GAPDH antibodies. *P < 0.5, **P < 0.01, ***P < 0.001, Error bars represent SEM. For comparisons between groups, Bonferroni's multiple comparison test was used. The western blot analysis presented; n= 2.

Fig. S6

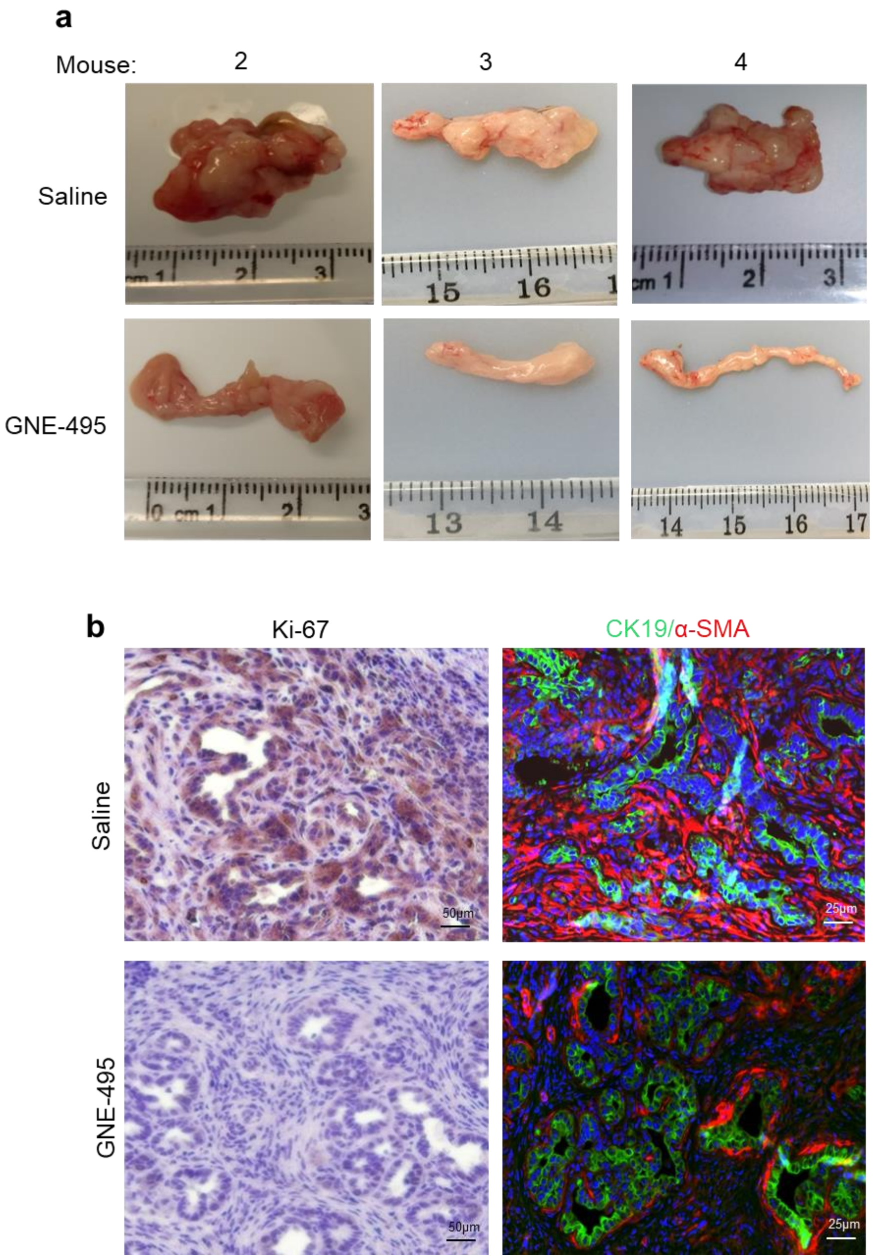

Supplementary Fig. 6 Pharmacological inhibition of MAP4K4 mitigates PDAC *in vivo*. The 2.5-month-old KPC mice were treated daily either with saline or GNE-495 (3 mg/kg body weight) until endpoint criteria. **(a)** The mouse pancreas was excised and weighed **(b)** The pancreatic tissues from saline and GNE-495 treated KPC mice were stained (either IHC or IF) with Ki67, CK19/αSMA, and pictures taken.
